# Künstliche Intelligenz (KI) in der Medizin: Erwartungen und Entwicklungen

**DOI:** 10.1007/s00103-025-04097-3

**Published:** 2025-07-23

**Authors:** Martin Sedlmayr, Joseph Kuhn, Wolfgang Lauer

**Affiliations:** 1 Institut für Medizinische Informatik und Biometrie & Zentrum für Medizinische Informatik, Medizinische Fakultät Carl Gustav Carus, TU Dresden, Fetscherstraße 74, 01309 Dresden, Deutschland; 2 Pettenkofer School of Public Health München, c/o Bayerisches Landesamt für Gesundheit und Lebensmittelsicherheit, Veterinärstr. 2, 85764 Oberschleißheim, Deutschland; 3 Abteilung Medizinprodukte, Bundesinstitut für Arzneimittel und Medizinprodukte (BfArM), Kurt-Georg-Kiesinger-Allee 3, 53175 Bonn, Deutschland

Künstliche Intelligenz (KI) hat auch im Gesundheitswesen schon seit Längerem Einzug gehalten, erlebt in den vergangenen Jahren aber dank zunehmender Digitalisierung, Interoperabilität und neuer technischer Möglichkeiten einen enormen Aufschwung. Die Nutzung von KI kann die Diagnostik, Behandlung und Verwaltung von Patient:innen revolutionieren, indem sie präzisere und schnellere Diagnosen ermöglicht, personalisierte Behandlungspläne unterstützt und administrative Prozesse optimiert. Dabei muss jedoch ein verantwortungsvoller und ethischer Umgang sichergestellt werden, um die Patientensicherheit zu gewährleisten und Datenschutz sowie ethische Standards einzuhalten.

Die Anwendungen von KI im Gesundheitswesen sind äußerst vielfältig. Ein großer Fokus liegt auf der Verbesserung der Diagnostik. Bildgebende Verfahren wie CT oder MRT profitieren von KI-Algorithmen, die in der Lage sind, aus den umfangreichen Daten schneller und oft genauer als menschliche Expert:innen Muster zu erkennen und Diagnosen zu unterstützen. In der Onkologie helfen KI-Systeme dabei, die Entwicklung von Tumoren präziser zu verfolgen und Therapieentscheidungen zu optimieren. Auch in der Genomik ermöglicht KI, genetische Dispositionen für Krankheiten schneller zu identifizieren und personalisierte Behandlungsansätze zu fördern.

Ein weiterer bedeutender Einsatzbereich ist das Management von Gesundheitsdaten. KI kann große Datenmengen analysieren, um Trends zu identifizieren, die Effizienz der Patientenversorgung zu steigern und die Ergebnisse zu verbessern. Dies reicht von der Vorhersage von Krankenhausaufenthalten bis hin zur Optimierung der Bettenbelegung und Ressourcenallokation. Darüber hinaus erleichtert KI die Fernüberwachung und Telemedizin, was besonders in ländlichen oder unterversorgten Gebieten von großer Bedeutung ist: beispielsweise indem Gesundheitsparameter bei Patient:innen über intelligente Geräte überwacht werden, die kontinuierlich Daten sammeln und bei Auffälligkeiten sofort Alarm schlagen.

Mit der Einführung, rasanten technologischen Weiterentwicklung und Ausweitung der Anwendung von KI sind aber auch Herausforderungen verbunden. Datenschutz ist hierbei ein zentrales Thema. Gesundheitsdaten sind besonders sensibel und der Schutz dieser Informationen vor Missbrauch und unerlaubtem Zugriff muss gewährleistet sein. Die Entwicklung von KI-Systemen erfordert daher nicht nur fortschrittliche technische Lösungen, sondern auch strenge ethische Richtlinien und angemessene gesetzliche Regelungen.

Ein weiteres Problem ist die Gefahr von Verzerrungen und Ungleichheiten, denn KI-Systeme lernen aus den Daten, die sie trainieren. Sind diese Daten unvollständig oder verzerrt, können die Schlussfolgerungen der KI fehlerhaft oder sogar diskriminierend sein. Dies erfordert eine sorgfältige Auswahl und Überprüfung der Trainingsdaten sowie kontinuierliche Tests der KI-Modelle und ihrer Ergebnisse auf Fairness, Genauigkeit und Erklärbarkeit.

Die Akzeptanz und das Vertrauen in KI-gestützte Systeme sind ebenfalls entscheidend und sowohl medizinisches Fachpersonal als auch Patient:innen müssen über die Funktionsweise, die Vorteile und die Grenzen der eingesetzten KI-Technologien aufgeklärt werden. Die Schulung des Personals spielt eine entscheidende Rolle, um sicherzustellen, dass diese Technologien effektiv genutzt werden, und ihre Vorteile zu maximieren.

Dort, wo KI zur Vorbereitung von Entscheidungen eingesetzt wird, z. B. bei der Zuweisung von Patient:innen zu bestimmten Behandlungspfaden, ist zudem das Verhältnis von KI-gestützten Datenanalysen und der Verantwortung von Entscheidungen und Entscheidenden zu reflektieren. Behandlungsentscheidungen müssen nachvollziehbar sein, ggf. auch vor Gericht. Dies wird auf absehbare Zeit zur Folge haben, dass eine KI autonom nicht über Behandlungsalternativen entscheiden kann. Eine Rückkehr zur „eminenzbasierten Medizin“ in Form eines blinden Vertrauens auf die KI soll vermieden werden. Zugleich werden aber KI-gestützte Systeme z. B. beim autonomen Fahren durchaus Entscheidungen über Leben und Tod treffen. Der Einsatz von KI im Gesundheitswesen ist also auch mit neuartigen ethischen Fragestellungen verbunden.

Der Einsatz von KI wird Arbeitsfelder im Gesundheitswesen tiefgreifend verändern und das betrifft bereits heute nicht nur „einfache“ Tätigkeiten, sondern auch hochqualifizierte Arbeit. Künstliche Intelligenz wird natürliche Intelligenz teilweise ersetzen, aber mit der Perspektive, an anderer Stelle mehr Menschlichkeit in der Medizin zu ermöglichen. Echtes Mitgefühl und menschliche Zuwendung werden absehbar nicht durch KI zu ersetzen sein.

Das Themenheft zielt darauf ab, die Breite der Thematik und darüber vermittelt die Dynamik des KI-Einsatzes im Gesundheitswesen aufzuzeigen: von den Grundlagen und Anwendungen der künstlichen Intelligenz über die relevanten Rahmenbedingungen bis hin zu exemplarischen Einsatzgebieten und Projekten. Dabei werden vor allem medizinische Anwendungen von KI fokussiert, auch wenn darüber hinaus noch viele weitere Anwendungen im Gesundheitswesen existieren, wie z. B. beim Einsatz von KI in der Psychotherapie, in der Prävention, bei der Entwicklung von neuen Medikamenten oder neuen Anwendungsindikationen, in der Synthese von Studienbefunden, im Medizinrecht, im Versicherungsmanagement, bei der Aufdeckung von Abrechnungsbetrug oder in der Praxisverwaltung – Anwendungsfälle, die im *Bundesgesundheitsblatt* in Zukunft immer wieder eine Rolle spielen werden. Der ethische Diskurs rund um den Einsatz von KI im Gesundheitswesen hat inzwischen erfreulicherweise an Breite gewonnen, konnte jedoch in diesem Rahmen nur kurz gestreift werden. Der Deutsche Ethikrat hat sich inzwischen wiederholt damit beschäftigt und ausdrücklich die Ambivalenz von Chancen und Risiken und damit verbunden den rechtlichen Regulierungsbedarf hervorgehoben (z. B. in seiner Stellungnahme „Mensch und Maschine – Herausforderungen durch Künstliche Intelligenz“ vom März 2023).

Beim Blick in die Zukunft fällt die Antwort der KI nicht anders aus als die der meisten menschlichen Beobachter. Ein bekannter Chatbot meint: „Der KI-Einsatz in der Medizin wird in den nächsten Jahren nicht nur punktuell, sondern strukturell das Gesundheitswesen verändern. Es ist realistisch, dass KI in nahezu allen Bereichen – von der Vorsorge über Diagnose und Therapie bis hin zur Nachsorge – eine entscheidende Rolle spielen wird.“ Das vorliegende Heft zeigt das exemplarisch. Wir wünschen den Leser:innen neue Einsichten und danken den Autor:innen für ihre Beiträge.

